# Beta1 integrin blockade overcomes doxorubicin resistance in human T-cell acute lymphoblastic leukemia

**DOI:** 10.1038/s41419-019-1593-2

**Published:** 2019-05-01

**Authors:** Sofiane Berrazouane, Marc Boisvert, Suzanne Salti, Walid Mourad, Reem Al-Daccak, Frédéric Barabé, Fawzi Aoudjit

**Affiliations:** 1 Division of Immune and Infectious Diseases, CHU de Québec-Université Laval Research Center, Quebec city, Canada; 2 CHUM Research Center and Faculty of Medicine, Université de Montréal, Montreal, Canada; 3 Hôpital St-Louis, INSERM Unit 976, Université Paris-Diderot, Paris, France; 4 Department of Medicine, Faculty of Medicine, Université Laval, Quebec city, Canada; 5 Department of Microbiology-infectious Diseases and Immunology, Faculty of Medicine, Université Laval, Quebec city, Canada

**Keywords:** Mechanisms of disease, Leukaemia

## Abstract

Growing evidence indicates that cell adhesion to extracellular matrix (ECM) plays an important role in cancer chemoresistance. Leukemic T cells express several adhesion receptors of the β1 integrin subfamily with which they interact with ECM. However, the role of β1 integrins in chemoresistance of T-cell acute lymphoblastic leukemia (T-ALL) is still ill defined. In this study, we demonstrate that interactions of human T-ALL cell lines and primary blasts with three-dimensional matrices including Matrigel and collagen type I gel promote their resistance to doxorubicin via β1 integrin. The blockade of β1 integrin with a specific neutralizing antibody sensitized xenografted CEM leukemic cells to doxorubicin, diminished the leukemic burden in the bone marrow and resulted in the extension of animal survival. Mechanistically, Matrigel/β1 integrin interaction enhanced T-ALL chemoresistance by promoting doxorubicin efflux through the activation of the ABCC1 drug transporter. Finally, our findings showed that Matrigel/β1 interaction enhanced doxorubicin efflux and chemoresistance by activating the FAK-related proline-rich tyrosine kinase 2 (PYK2) as both PYK2 inhibitor and siRNA diminished the effect of Matrigel. Collectively, these results support the role of β1 integrin in T-ALL chemoresistance and suggest that the β1 integrin pathway can constitute a therapeutic target to avoid chemoresistance and relapsed-disease in human T-ALL.

## Introduction

Acute T cell lymphoblastic leukemia (T-ALL) accounts for approximately 15% of all childhood acute leukemia and 25% of adult ALLs. Although chemotherapy has improved the treatment of T-ALL, the prognosis of drug resistant and relapsed T-ALL remains very poor^1^. Thus, defining the factors promoting T-ALL chemoresistance is critical to improve this prognosis.

The major development site for T-ALL is the bone marrow where the osteoblastic (endosteal) and vascular niches regulate the fate of normal and transformed hematopoietic cells^2,3^. These two niches are rich in adhesive substrates particularly the extracellular matrix (ECM). Normal and malignant hematopoietic cells express several adhesion receptors among which, is the β1 integrin subfamily that mediates their interactions with stromal cells and ECM. The β1 integrins are α/β heterodimeric membrane receptors, which upon ligand binding induce activation of non-receptor tyrosine kinases including focal adhesion kinase (FAK) and its homologous FAK-related proline-rich tyrosine kinase 2 (PYK2) leading to cytoskeletal rearrangements and downstream signaling^4^.

Cell adhesion to stromal cells and ECM plays an important role in the development of chemoresistance^5–7^. In hematological malignancies, the integrin α4β1, upon binding to its counter-receptor, the vascular cell adhesion molecule-1 or to fibronectin, has been implicated in the chemoresistance of myeloma^8–10^ and B cell malignancies^11–13^ whereas, binding to fibronectin through integrins α4β1 and α5β1 has been associated with the chemoresistance of myeloid leukemia^14–16^.

T-ALL cells express several β1 integrin receptors with which they interact with various ECM proteins including collagens, fibronectin and laminins^17,18^. In this context, collagen type I, *via* α2β1 integrin, has been shown to promote T-ALL chemoresistance^19^. Similarly, crosslinking of α4β1 and α5β1 integrins with recombinant fibronectin-derived ligands equally enhances T-ALL chemoresistance^20^. Both fibronectin and collagen type I are enriched in the endosteal niche of the bone marrow^21^. However, T-ALL cells also interact with the vascular niche^22,23^, which is enriched in laminins and collagen type IV, but the role of the vascular niche in T-ALL chemoresistance has not been determined.

The above studies on T-ALL chemoresistance were conducted with two-dimensional (2D) matrix models whereas the cells in their niches are likely interacting with a three-dimensional (3D)-organized matrix, which has different signaling properties than the 2D matrix models, raising the issue of whether β1 integrin-mediated chemoresistance could be recapitulated with a 3D matrix. In addition, it remains undetermined if targeting β1 integrin could improve chemotherapy and constitutes a therapeutic target in T-ALL.

In this study, we found that attachment to Matrigel, a 3D matrix model mimicking ECM of the vascular niche, promotes T-ALL chemoresistance via β1 integrin. In addition, β1 integrin blockade sensitized xenografted leukemic cells to chemotherapy and resulted in prolonged animal survival. Finally, our results showed that β1 integrin enhanced chemoresistance by activating drug efflux in a PYK2-dependant manner. Collectively our findings suggest that the β1 integrin pathway could represent a new therapeutic target to avoid chemoresistance and relapsed-disease in human T-ALL.

## Results

### Matrigel protects T-ALL cell lines from doxorubicin-induced apoptosis

**Fig. 1:**
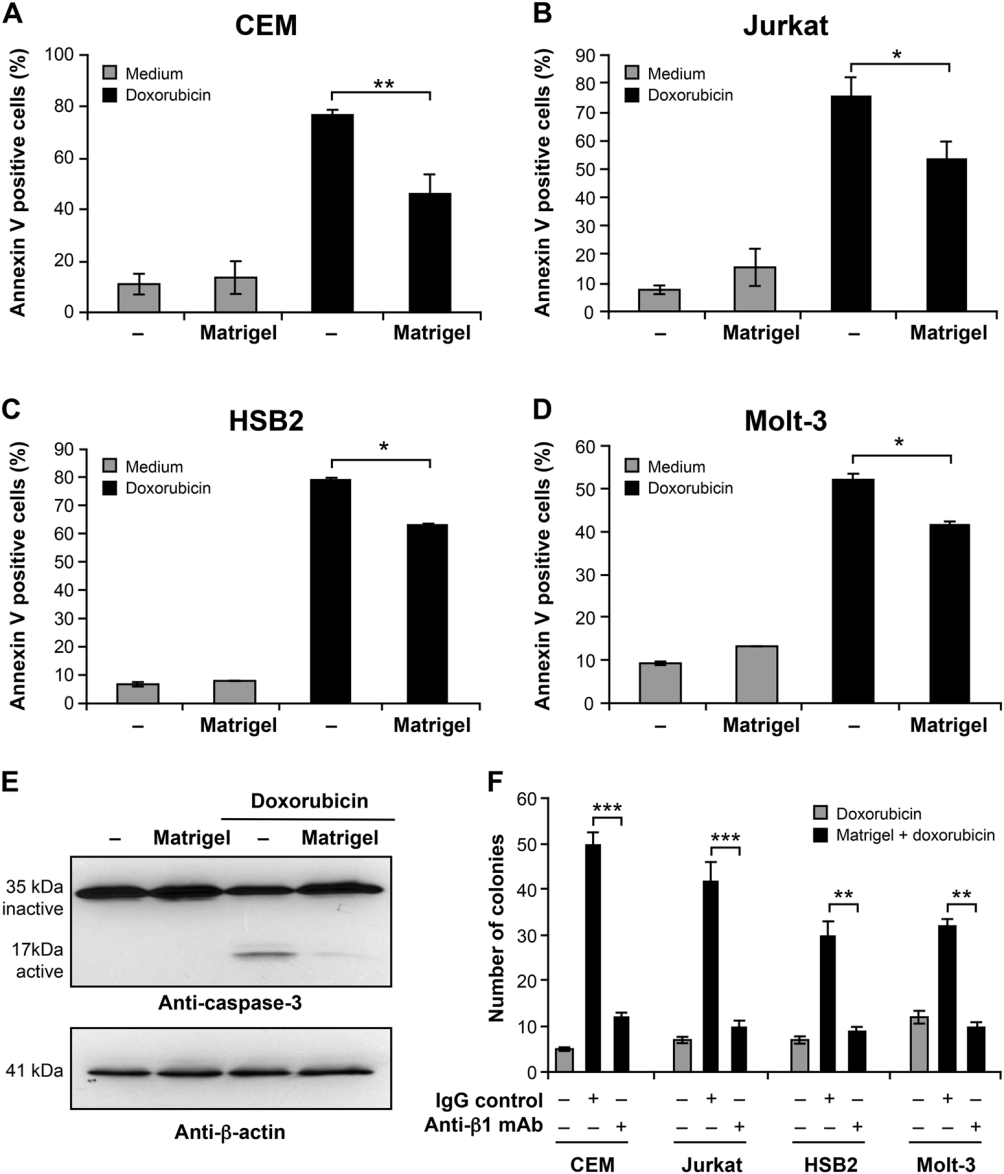
Attachment to Matrigel promotes doxorubicin resistance of T-ALL cell lines through β1 integrin. CEM **a**, Jurkat **b**, HSB-2 **c**, Molt-3 **d** were cultured on plastic (−) or on Matrigel for 4 h and then treated or not with doxorubicin. After 24 h, apoptosis was analyzed by annexin V staining and flow cytometry. **e** Matrigel inhibits doxorubicin-induced caspase-3 activation. CEM cells were cultured on Matrigel or on plastic (−) and then treated or not with doxorubicin for 12 h. Cells were lysed and cell lysates subjected to immunoblot analysis with an anti-caspase-3 antibody. The blot was stripped and reprobed with anti-β-actin antibody for equal loading. The blot is representative of three independent experiments. **f** Matrigel promotes clonogenic growth via β1 integrin. Clonogenic growth of T-ALL cell lines was determined in the presence of 10 μg/ml of control IgG or anti-human β1 integrin blocking mAb (AIIB2), which were added before seeding the cells on Matrigel. Results represent the mean values ± S.D. of three independent experiments. *P < 0.05; **P < 0.01; ***P < 0.001

To examine the implication of the ECM present in the vascular niche and the role of a 3D matrix in T-ALL chemoresistance, we studied the effect of Matrigel on drug-induced apoptosis in human T-ALL cell lines (CEM, Jurkat, HSB2 and Molt-3), which express variable levels of α integrins and high levels of the β1 integrin chain^17^. Attachment of various T-ALL cell lines to Matrigel reduced their apoptosis induced upon exposure to doxorubicin (Fig. 1a–d). The best inhibitory effect was observed in CEM and Jurkat T cell lines where drug-induced apoptosis is reduced by 30–40%. To confirm the anti-apoptotic effect of Matrigel, we determined its effect on doxorubicin-induced caspase-3 activation, which is a main apoptotic event in drug-induced apoptosis. The results show that doxorubicin activates caspase-3 as determined by the proteolysis of procaspase-3 and the appearance of active caspase-3 fragments, and culture of CEM cells on Matrigel significantly reduced doxorubicin-induced caspase-3 activation (Fig. 1e).

We then conducted clonogenic survival assays to evaluate if Matrigel promoted long-term survival. As shown, treatment of the cells with doxorubicin led to the formation of very few colonies (Fig. 1f). However, the presence of Matrigel, which inhibited doxorubicin-induced apoptosis, led in the four T cell lines, to a remarkable increase in the number of colonies compared to doxorubicin-treated samples. In line with the apoptosis data, the number of colonies induced by Matrigel is higher in CEM and Jurkat than in Molt-3 and HSB2 T cell lines (Fig. 1f). To assess if the role of Matrigel is mediated via β1 integrins, we tested the effect of the well-characterized human β1 integrin-specific blocking mAb, AIIB2, which has been shown to block tumor cell-interactions with 3D matrices including Matrigel^24,25^. Addition of AIIB2 substantially reduced the Matrigel effect on colony formation (Fig. 1f), indicating the implication of β1 integrin in chemoresistance. In addition to Matrigel, β1 integrin also mediates the protective effect of collagen type I gel; an additional 3D matrix model (Supplementary Fig. 1).

### Matrigel protects primary T-ALL blasts from doxorubicin-induced apoptosis

**Fig. 2:**
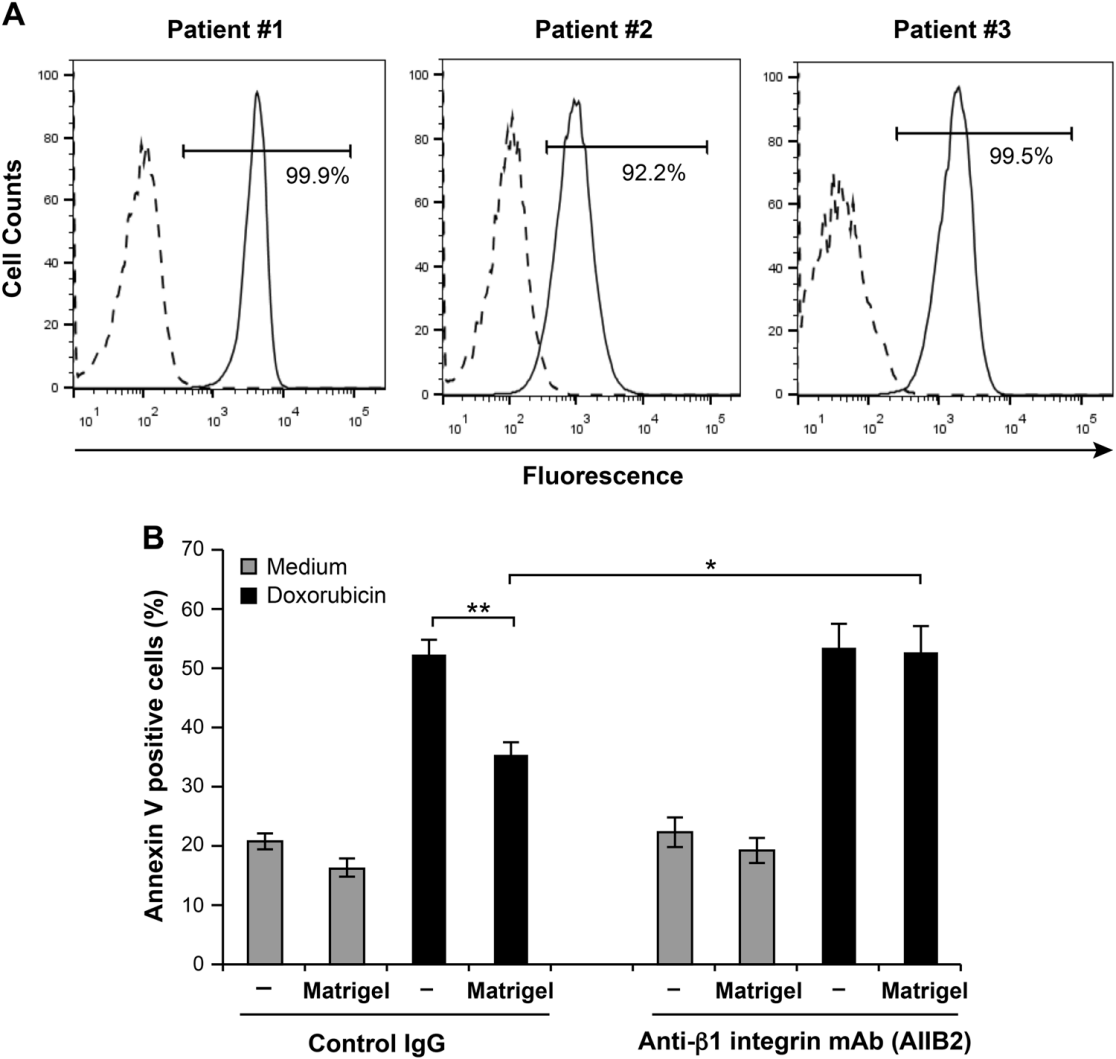
Matrigel/β1 integrin promotes doxorubicin resistance of primary T-ALL blasts. **a** Flow cytometry expression of β1 integrin chain on three different T-ALL blasts. The percentage of positive cells is indicated. **b** β1 integrin mediates the protective effect of Matrigel. Primary T-ALL blasts were cultured on Matrigel in the presence of 10 μg/ml of control IgG or anti-β1 integrin blocking mAb (AIIB2) and then treated for 24 h with doxorubicin. Apoptosis was analyzed by annexin V staining and flow cytometry. Results represent the mean values ± S.D. obtained with blasts from three different T-ALL patients. *P < 0.05; **P < 0.01

To determine if the findings with T-ALL cell lines occur in a clinical setting, we assessed the expression of β1 integrin and the effect of Matrigel in primary T-ALL blasts. Three different T-ALL samples expressing surface CD3 originating from the blood of patients at diagnosis were obtained and analyzed for their expression of β1 integrin. β1 integrin is highly expressed in all samples; between 92 and 99% of total cells expressed β1 (Fig. 2a). Similar to T-ALL cell lines, attachment to Matrigel reduced doxorubicin-induced apoptosis in T-ALL blasts (Fig. 2b). Importantly, the protective effect of Matrigel was reversed by the β1 integrin blocking mAb AIIB2 (Fig. 2b). As a control, AIIB2 had no effect on the survival of cells cultured in medium or in the presence of Matrigel. Thus, 3D matrices promote doxorubicin resistance of both human T-ALL cell lines and primary T-ALL blasts via β1 integrin.

### Blockade of β1 integrin sensitizes human T-ALL xenograft to chemotherapy

To examine whether the role of β1 integrin is relevant in vivo, we tested the effect of the human β1 integrin-specific blocking mAb, AIIB2, in the human T-ALL CEM xenograft model^26,27^. In addition, AIIB2 has been shown to block β1 integrin in various tumor cell models in vivo^24,25^.

**Fig. 3:**
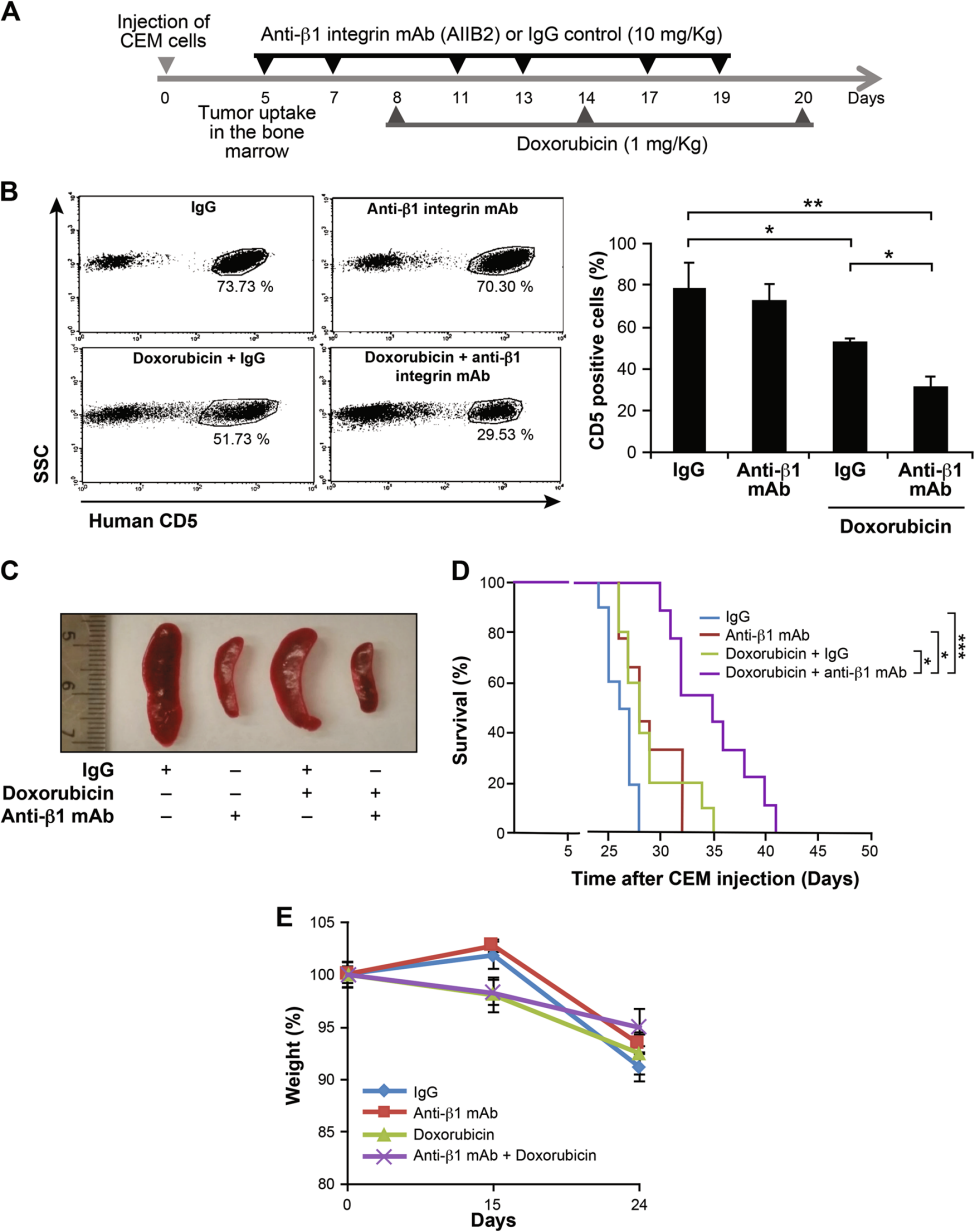
β1 integrin promotes T-ALL resistance to doxorubicin in vivo. **a** Scheme of animal protocol. **b** The blocking anti-human β1 integrin mAb (AIIB2) sensitizes xenotransplanted CEM cells to doxorubicin. Leukemic mice were treated as indicated, and at day 19, bone marrow cells were recovered from sacrificed mice, stained with PE-conjugated anti-human CD5 mAb and analyzed by flow cytometry. Representative FACS profiles (left panel) showing numbers of CD5^+^ cells in the bone marrow. The histogram (right panel) represents quantification of CD5^+^ cells numbers in the bone marrow (*n* = 6 mice/group). **c** Representative spleens recovered from leukemic mice at day 28. **d** Kaplan-Meyer analysis of animal survival in the different experimental groups (*n* = 9-10 per group). **e** Variation in animal weights across the four treatment groups. Results represent the mean values ± S.D. of two independent experiments. *P < 0.05; **P < 0.01; ***P < 0.001

Thus, we first assessed leukemia growth by flow cytometry using the marker CD5, which is highly expressed on CEM cells^26,27^. Mice-bearing leukemia were treated with control IgG, AIIB2, IgG + doxorubicin or AIIB2 + doxorubicin (Fig. 3a). Treatment with AIIB2 alone had no effect on the number of leukemic cells (CD5^+^) in the bone marrow (Fig. 3b). However, doxorubicin in the presence of a control IgG reduced leukemic load by approximately 30% (Fig. 3b). More importantly, the addition of AIIB2 significantly enhanced the effect of doxorubicin leading to 60% reduction in leukemic cell numbers compared to control IgG-treated mice (Fig. 3b). In contrast to the bone marrow, AIIB2 alone and in combination with doxorubicin inhibited tumor growth in the spleen (Fig. 3c).

We then examined whether treatment with AIIB2 will result in the extension of animal survival. Treatment of mice with AIIB2 alone had a marginal and a non-significant effect (*P* *=* 0.3) on their survival time compared to control IgG-treated mice (Fig. 3d). Similarly, treatment with doxorubicin also had a minor effect compared to control animals but was not significant (*P* *=* 0.3). However, treatment with doxorubicin + AIIB2 significantly extended the survival of leukemic mice compared to other treatment groups (Fig. 3d). The mean survival time of the doxorubicin + AIIB2 group is 34.8 *vs* 26.2 days for the control IgG group (*P* < 0.001). Interestingly, all animals in the doxorubicin + AIIB2 group had their survival time prolonged.

Treatment with AIIB2 did not affect weight loss of leukemic mice (Fig. 3e) suggesting that it did not exacerbate the burden of leukemia or the toxic effect of doxorubicin.

Finally, since AIIB2 might cross-react with mouse β1 integrin, we sought to determine whether its sensitizing effect was due to its action on β1 integrin expressed by mouse bone marrow cells. To test this possibility, we assessed the effect of a mouse β1 integrin blocking mAb (Ha2/5). The results showed that treatment of leukemic mice with this antibody alone and in combination with doxorubicin had no effect on animal survival (Supplementary Fig. 2) suggesting that it did not sensitize xenografted leukemic cells to the effect of doxorubicin. Taken together, these results indicate that targeting β1 integrin overcomes doxorubicin resistance of leukemic cells in vivo by acting on leukemic cells.

### Matrigel/β1 integrin enhances doxorubicin efflux

**Fig. 4:**
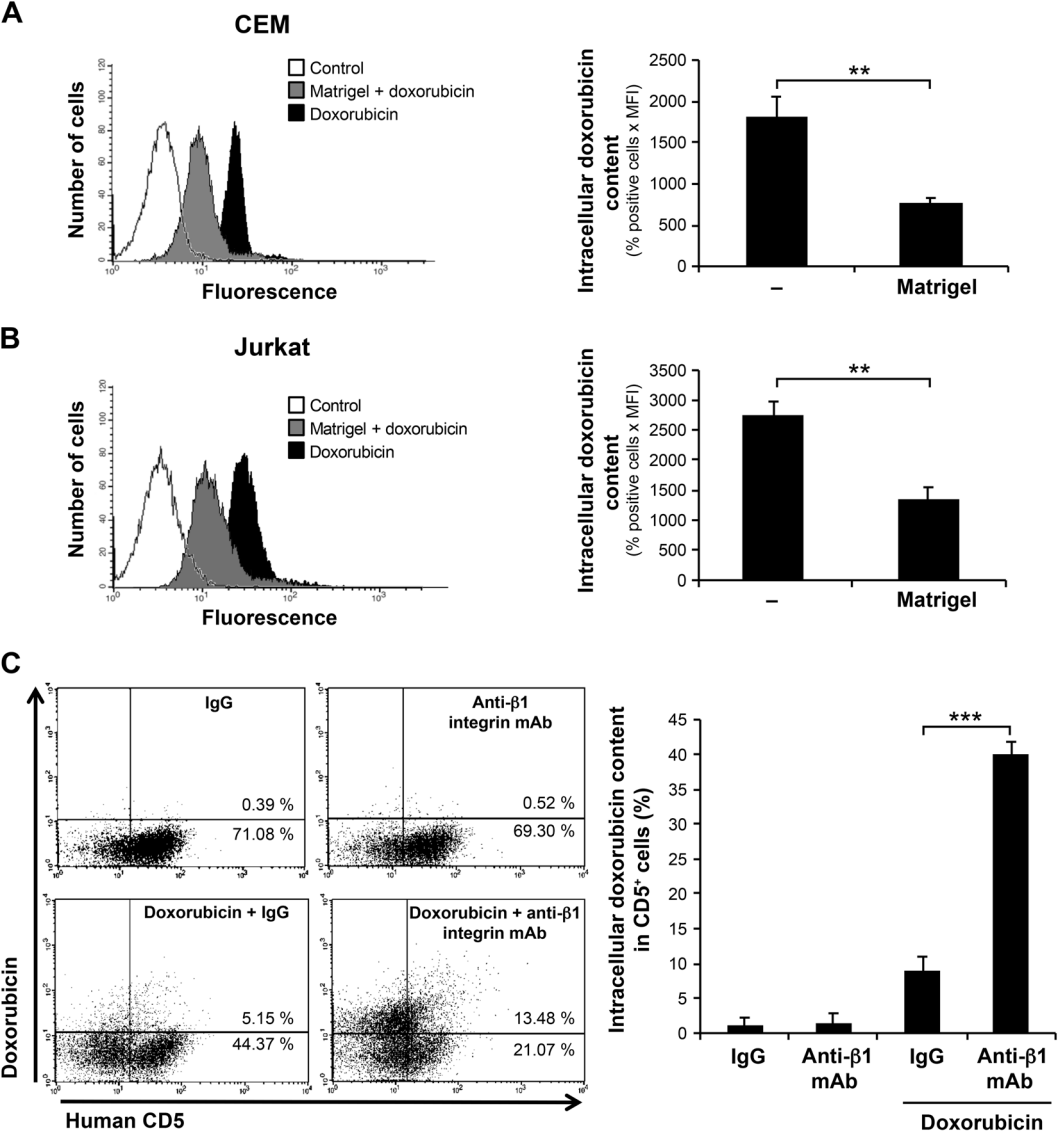
Matrigel/β1 integrin promotes doxorubicin efflux. Matrigel reduces doxorubicin intracellular content in human T-ALL cell lines. CEM **a** and Jurkat **b** were cultured on Matrigel or on plastic (−) for 4 h and then treated with doxorubicin for 2 h. The cells were washed and analyzed by flow cytometry. Representative FACS profiles (left panels) showing intracellular doxorubicin content. The histograms (right panels) represent quantification of intracellular doxorubicin content. **c** Blockade of β1 integrin with the anti-β1 integrin mAb (AIIB2) increases intracellular doxorubicin content in xenotransplanted CEM cells. Leukemic mice were treated as indicated and sacrificed one day after the last injection of doxorubicin (day 21). Bone marrow cells were recovered from leukemic mice (*n* = 6), stained with Alexa-647 conjugated anti-human CD5 mAb and analyzed by flow cytometry. Representative FACS profiles (left panel) showing intracellular doxorubicin content. The histogram (right panel) represents quantification of intracellular doxorubicin content in the CD5^+^ cell population. Results represent the mean values ± S.D. from two (panel C, right panel) and three (panels A&B) independent experiments. **P < 0.01; ***P < 0.001

After demonstrating the role of β1 integrin in T-ALL chemoresistance, we examined the mechanisms by which β1 integrin promotes chemoresistance. One major mechanism accounting for drug resistance in cancer cells is the decrease in intracellular drug concentrations *via* the activation of drug efflux, which is mediated by several membrane drug transporters that belong to the ATP-binding cassette (ABC) superfamily^28^. To test this possibility, we first assessed if Matrigel would reduce intracellular doxorubicin content. The results show that the culture of T-ALL cell lines on Matrigel reduces by 60% the intracellular doxorubicin content in CEM and Jurkat cells (Fig. 4a, b). We then determined if a similar mechanism occurs in vivo upon treatment with the β1 integrin blocking mAb AIIB2. Mice were treated with doxorubicin and antibodies, sacrificed at day 21, and bone marrow cells were harvested one day after treatment with doxorubicin. We found that leukemic cells (CD5^+^) harvested from bone marrow of mice treated with doxorubicin + control IgG had a low level of intracellular doxorubicin as only 10% of the leukemic cells were fluorescent (Fig. 4c). However, treatment of mice with doxorubicin + AIIB2 led to approximately 40% of leukemic cells that contain doxorubicin (Fig. 4c) indicating that blocking β1 integrin in vivo sensitized the cells towards more doxorubicin accumulation. Similar results were obtained when quantification of intracellular doxorubicin content was expressed as (% positive cells × MFI) (Supplementary Fig. 3).

### ABCC1 mediates Matrigel/β1 integrin-induced doxorubicin efflux and resistance in T-ALL cells

**Fig. 5:**
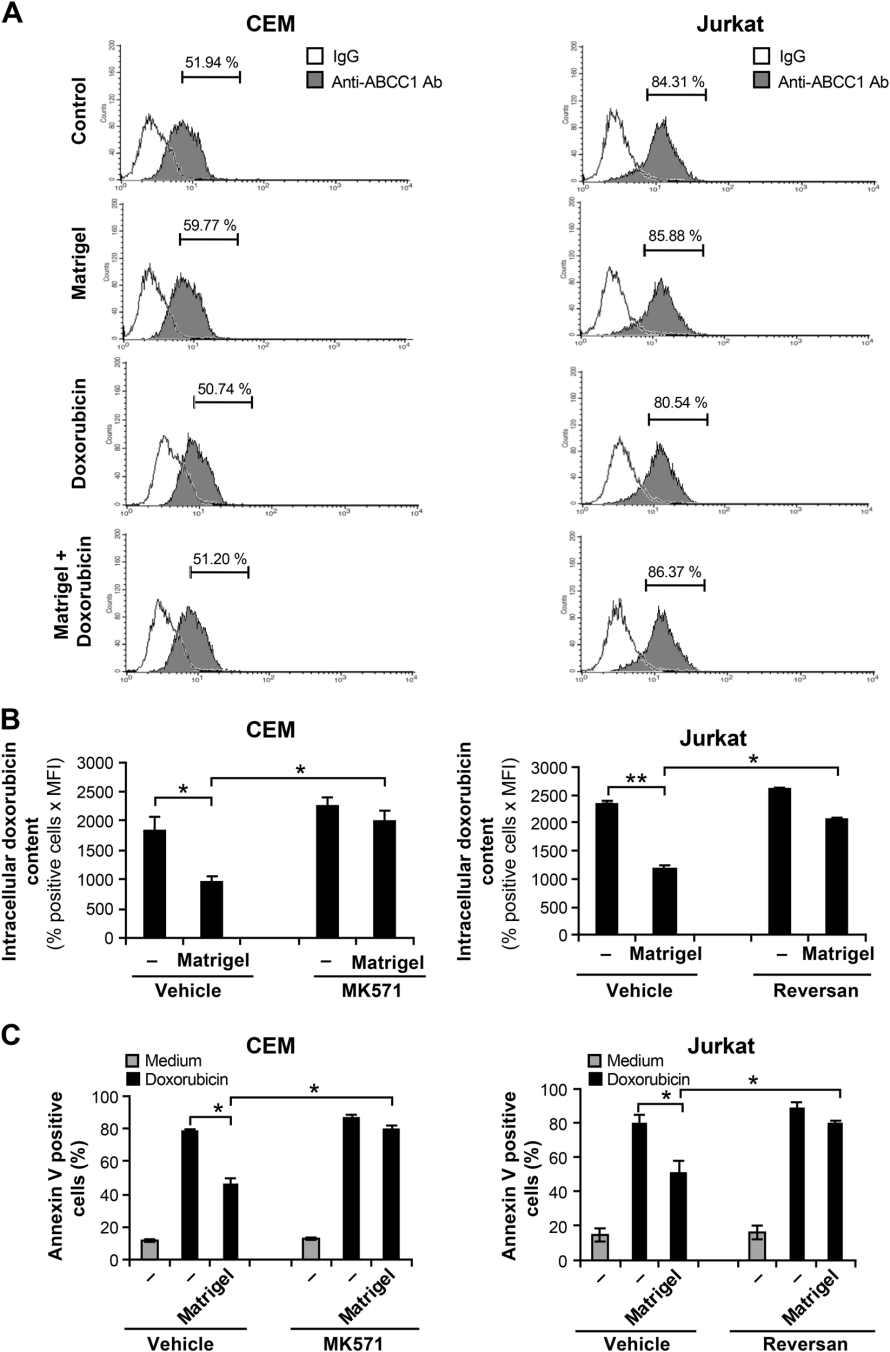
Matrigel/β1 integrin promotes doxorubicin resistance *via* ABCC1. **a** Expression levels of ABCC1 in CEM and Jurkat T cell lines as determined by flow cytometry (% of positive cells is indicated). **b** ABCC1 inhibitors MK571 and reversan inhibit the ability of Matrigel to induce doxorubicin efflux. The cells were pre-treated with the vehicle as control or with 10 μM of MK571 or reversan for 1 h and then cultured on plastic (−) or on Matrigel for 4 h. The cells were then treated for 2 h with doxorubicin and the content of intracellular doxorubicin was determined by flow cytometry analysis. **c** The cells were pre-treated with inhibitors, cultured on Matrigel and treated with doxorubicin as above. Apoptosis was analyzed by annexin V staining and flow cytometry after 24 h of treatment with doxorubicin. Results represent the mean values ± S.D. from three independent experiments. *P < 0.05; **P < 0.01

There are two ABC transporters namely ABCB1 (Pg-P) and ABCC1 (MRP-1) that are known to play a major role in doxorubicin efflux. Previous studies have shown that human T-ALL cell lines express ABCC1 but not ABCB1^29,30^. In agreement, we did not detect the expression of ABCB1 and Matrigel or doxorubicin did not upregulate ABCB1 levels (data not shown). However, we found that around 50% of CEM and 80% of Jurkat cells express ABCC1 (Fig. 5a). The results also showed that ABCC1 levels in CEM and Jurkat T cells lines were not significantly affected by Matrigel or by doxorubicin.

To examine whether ABCC1 is involved in doxorubicin efflux, we evaluated the effect of two specific ABCC1 inhibitors namely MK571 and reversan. The results show that both inhibitors almost completely abolished the effect of Matrigel on intracellular doxorubicin content (Fig. 5b) and the protective effect of Matrigel on doxorubicin-induced apoptosis (Fig. 5c). For further support, we studied the effect of Matrigel on vincristine, a known substrate of ABCC1. Matrigel also reduced vincristine-induced apoptosis in CEM T cell line and the ABCC1 inhibitor MK571 reversed this effect suggesting the implication of ABCC1 (Supplementary Fig. 4). Together these data indicate that ABCC1 plays a major role in β1 integrin-induced drug efflux and chemoresistance in human T-ALL cells.

### Matrigel/β1 integrin promotes doxorubicin resistance via PYK2

**Fig. 6:**
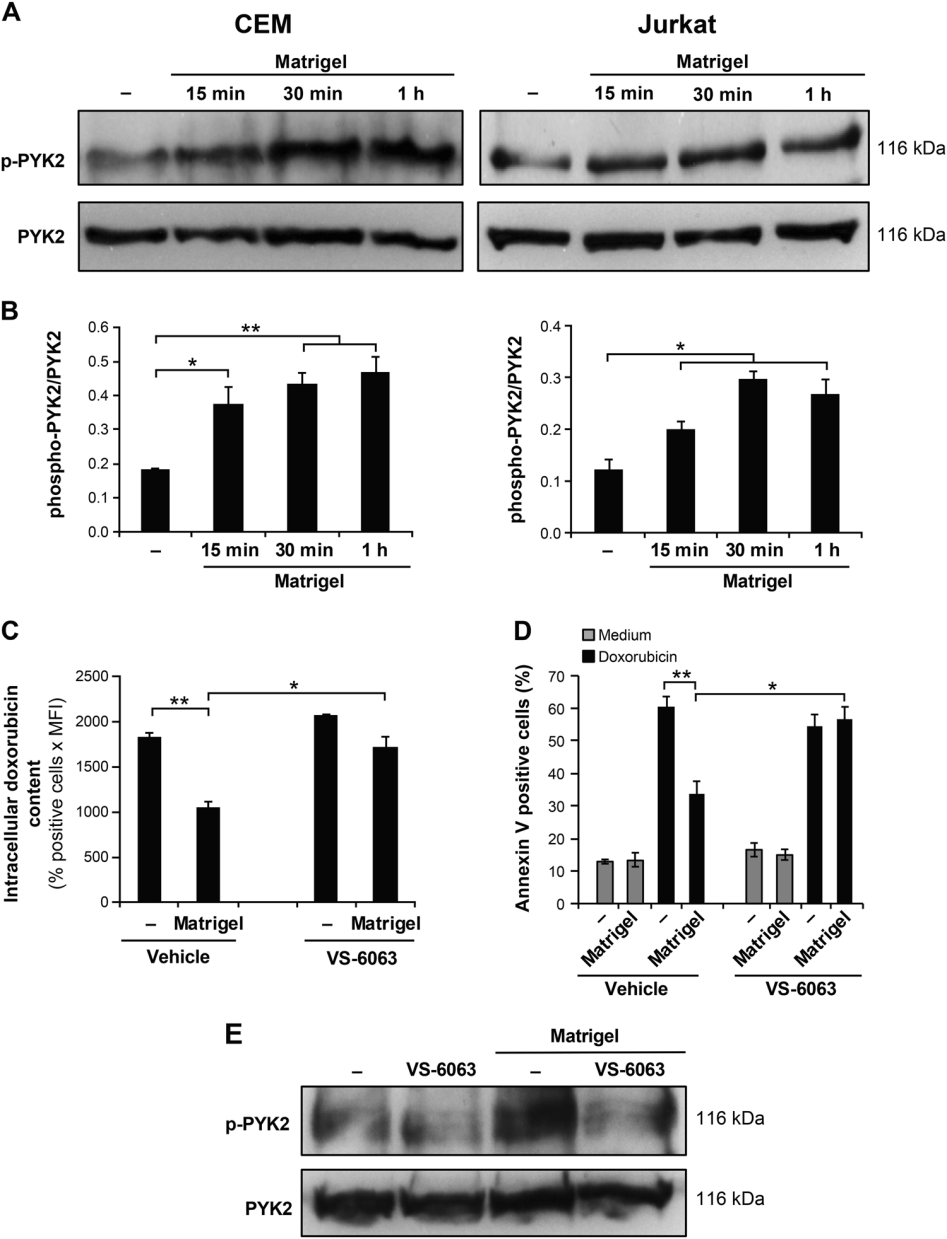
Matrigel/β1 integrin-mediated doxorubicin resistance is dependent on PYK2. **a** Attachment to Matrigel increases PYK2 activation. CEM and Jurkat cells were cultured on Matrigel for the indicated periods of time. The cells were lysed and cell lysates subjected to immunoblot analysis using anti-phosphorylated PYK2 (Tyr-402) antibody. The blots were stripped and reprobed with anti-PYK2 antibody for equal loading. **b** Histograms represent densitometric quantification of PYK2 activation. The results are expressed as ratios between phosphorylated PYK2 (p-PYK2) and total PYK2. **c** The PYK2 inhibitor VS-6063 inhibits the effect of Matrigel in CEM T-ALL cell line. CEM cells were pre-treated for 1 h with the vehicle or with 0.5 μM of VS-6063, cultured on plastic (−) or on Matrigel for 4 h and then treated with doxorubicin for 2 h. Intracellular doxorubicin content was then determined by flow cytometry. **d** CEM cells were pre-treated with VS-6063, cultured on Matrigel and then treated with doxorubicin for 24 h. Apoptosis was then analyzed by annexin V staining and flow cytometry. **e** VS-6063 inhibits Matrigel-induced PYK2 activation in CEM cells. The blot is representative of three independent experiments. Results represent the mean values ± S.D. of three independent experiments. *P < 0.05; **P < 0.01

Growing evidence suggests that the related-focal adhesion kinase, PYK2, which is known to be downstream of integrins and associated with T cell signaling, plays a major role in cancer. Accordingly, we studied its implication in cell adhesion-mediated T-ALL chemoresistance. We found that Matrigel increased by 2 to 3 fold the phosphorylation of PYK2 in both CEM and Jurkat T-ALL cell lines (Fig. 6a, b). PYK2 phosphorylation was detected after 15 min and peaked between 30 min to 1 h.

We then investigated the implication of PYK2 in the observed chemoresistance by determining the effect of a dual PYK2/FAK inhibitor, VS-6063 in CEM cells. We found that VS-6063 inhibited the effect of Matrigel on doxorubicin efflux as the intracellular doxorubicin content in Matrigel-activated CEM cells cultured in the presence of VS-6063 was similar to that observed in cells cultured on plastic (Fig. 6c). Furthermore, VS-6063 also inhibited the ability of Matrigel to reduce doxorubicin-induced apoptosis (Fig. 6d). However, VS-6063 has not affected the survival of CEM cells cultured in medium or in the presence of Matrigel. As expected, VS-6063 inhibited Matrigel-induced PYK2 activation in CEM cells (Fig. 6e). Collectively these results suggest that PYK2 is critical for Matrigel-induced doxorubicin resistance in T-ALL cells.

**Fig. 7:**
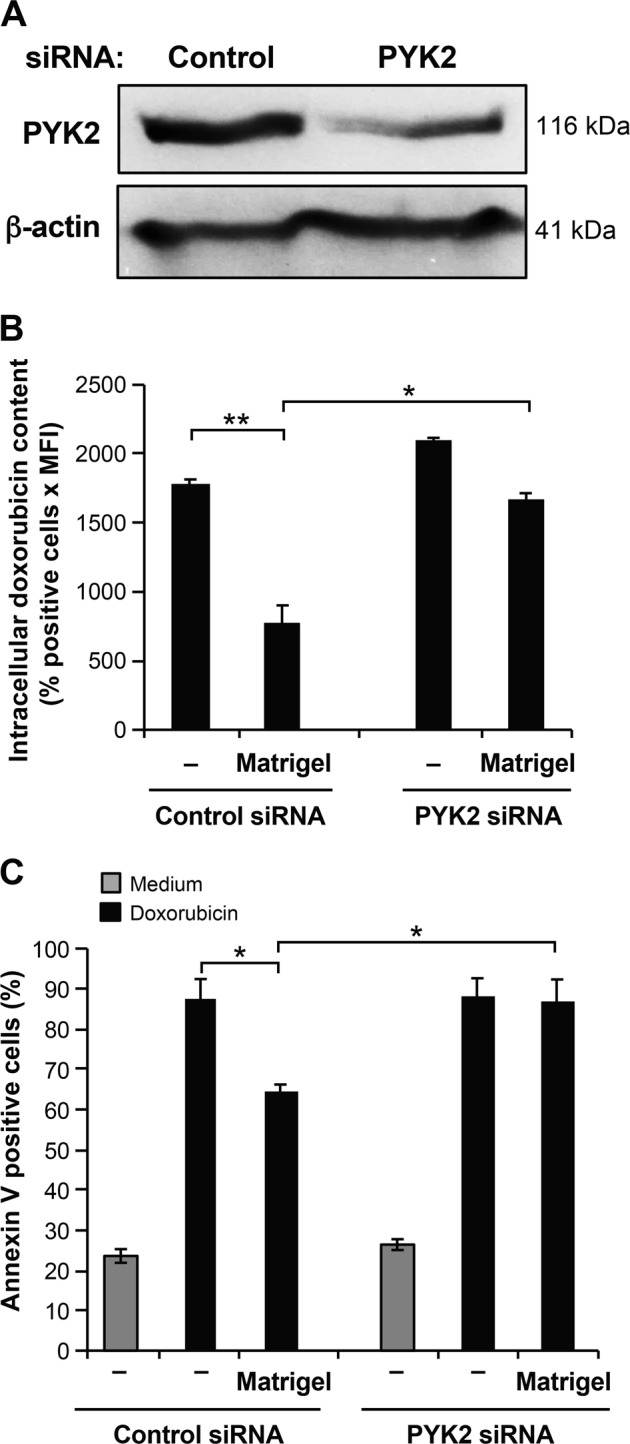
PYK2 siRNA abolishes the protective effect of Matrigel. **a** Jurkat cells were transfected with control and PYK2 siRNAs. After transfection, the expression of PYK2 protein was determined in control and in PYK2 siRNA-transfected cells by immunoblot analysis. β-actin levels were determined as a control to ensure equal loading. **b** Intracellular doxorubicin content in control and in PYK2 siRNA-transfected cells was assessed by flow cytometry analysis. **c** Apoptosis of Jurkat cells transfected with control and PYK2 siRNA was analyzed by annexin V staining and flow cytometry. Results represent the mean values ± S.D. of three independent experiments. *P < 0.05; **P < 0.01

Since PYK2 inhibitors could have off-target effects, we wished to confirm the implication of PYK2 by performing a PYK2 knockdown. Transfection of Jurkat T cells with a PYK2 specific siRNA reduced the levels of PYK2 compared to control siRNA-transfected Jurkat T cells (Fig. 7a) (approximately 65–70% reduction; average of three experiments). Importantly, compared to control siRNA, PYK2 siRNA significantly inhibited the ability of Matrigel to reduce intracellular doxorubicin content (Fig. 7b) and to protect Jurkat T cells from doxorubicin-induced apoptosis (Fig. 7c). These results indicate that Matrigel/β1 integrin promotes doxorubicin chemoresistance *via* PYK2.

## Discussion

Understanding the mechanisms underlying cancer chemoresistance is likely to lead to more effective therapies and to a better control of patient relapse. In this study, we report that blockade of the β1 integrin cell adhesion molecule overcomes T-ALL doxorubicin resistance by activating its efflux via PYK2.

Our results showed that T-ALL cells are capable of interacting *via* β1 integrins with 3D matrix proteins Matrigel and collagen type I gel to enhance their resistance to chemotherapy. Matrigel, which contains laminins, collagen IV and proteoglycans mimics the ECM present in the vascular niche, whereas collagen type I is enriched in the endosteal niche. It has been reported that during T-ALL progression, leukemic cells engage in dynamic interactions with bone marrow microenvironment components present in both the endosteal and vascular niches^31^. This is in line with our results and indicates that both niches can provide signals *via* β1 integrins to support T-ALL chemoresistance. Although we were limited in the number of primary T-ALL blasts, our results showed that Matrigel-β1 integrin-mediated chemoresistance also occurs in human T-ALL.

The blockade of β1 integrin with the specific neutralizing mAb AIIB2 sensitized CEM-xenografted cells to doxorubicin and resulted in the extension of animal survival. Although doxorubicin reduced bone marrow tumor load by 30%, it had only a minor effect on animal survival suggesting that xenotransplanted CEM cells have acquired resistance to doxorubicin and the observed reduction in leukemic load is not sufficient to alleviate the leukemia burden. This finding is in line with a previous study, which reported resistance of CEM cells to chemotherapy when transplanted in immunocompromised mice^27^. However, treatment with AIIB2 increased the effect of doxorubicin, further diminished bone marrow tumor load and extended animal survival indicating that β1 integrin plays a major role in doxorubicin resistance. In contrast to chemoresistance, we found that inhibition of β1 integrin with AIIB2 did not affect leukemic growth in the bone marrow but did so in the spleen. The role of β1 integrins in T-ALL development and progression is still poorly addressed but this could be explained by the capacity of β1 integrin to differentially sustain T-ALL growth in different hematologic sites or by the known role of β1 integrin in metastasis and invasion of malignant T cells^32,33^. In this regard, α4β1 integrin is essential for T cell transendothelial migration and has been involved in T cell lymphoma metastasis to the spleen and other secondary lymphoid tissues^34^. Despite this effect, blockade of β1 integrin had only a marginal effect on animal survival, which is likely due to the bone marrow disease that is not inhibited by the β1 integrin blocking mAb AIIB2, indicating that β1 integrin is dispensable for T-ALL growth in the bone marrow. Together these results propose an important role for β1 integrin in T-ALL chemoresistance, which is supported by a clinical study showing that cell adhesion receptors of the β1 integrin subfamily are the best predictors of patient relapse in pediatric T-ALL^35^. Thus, β1 integrin is likely to constitute a major pathway and target in T-ALL chemoresistance.

Mechanistically, we found that β1 integrin enhances T-ALL chemoresistance by activating drug efflux *via* the drug transporter ABCC1. This was shown both in vitro, and in vivo with the CEM xenograft model. These findings are likely to be of clinical significance since Matrigel/β1 integrin protects T-ALL blasts from doxorubicin (Fig. 2) and that ABCC1 is expressed in human T-ALL cells and its activity correlates with poor prognosis^36,37^.

β1 integrin enhanced drug efflux not only in xenotransplanted CEM leukemic cells but also in mouse bone marrow cells (Fig. 4c). We did not investigate the identity of these cells but a recent study reported that β1 integrin is important for the acquisition of side population (drug efflux and resistance to drugs) by hematopoietic stem progenitor cells^38^ suggesting that β1 could be important for drug resistance of leukemia initiating cells *via* the activation of ABC transporters. Along these lines, β1 integrin enhances mitoxantrone efflux in AML blasts upon their culture with mesenchymal stem cells^38^. Interestingly, mitoxantrone is a substrate for the drug transporter, ABCG2, whereas doxorubicin is a substrate for ABCC1. Thus, together these studies strongly suggest that β1 integrin-mediated activation of ABC transporters can be a major pathway in drug resistance of hematological malignancies.

We found that Matrigel did not increase ABCC1 expression levels indicating that its effect occurs likely at the activation level. In this regard, it has been shown that actin polymerization is required for the localization, stability and function of ABCC1^39^. Accordingly, in our cell model, T-ALL cell interaction with Matrigel can lead to β1 integrin-mediated actin polymerization, which then enhances ABCC1 activity. The mechanisms involved in ABCC1 activity are not well understood and factors such as receptor cycling and increased ATP production, which are required for the function of ABC transporters, could also be involved in β1 integrin-mediated ABCC1 activation.

Finally, we showed that β1 integrin-mediated doxorubicin efflux and resistance in T-ALL cells involves PYK2. PYK2 is a member of the FAK family tyrosine kinase and has been associated with T cell signaling and cell migration downstream of integrins^40^. Our results indicate that inhibition of PYK2 reverses the effect of Matrigel on doxorubicin efflux and chemoresistance. A recent study also showed the implication of PYK2 in IL-6-dependant chemoresistance of ovarian cancer cells^41^. Thus, these studies suggest that PYK2 could play a crucial role in the tumor microenvironment-mediated cancer chemoresistance. The uncovered implication of PYK2 in T-ALL chemoresistance coupled with its reported role in myeloma cell survival and progression^42–44^ suggest that PYK2 could represent an important target in hematological malignancies.

In summary, we report the first study to our knowledge, indicating that targeting β1 integrin could constitute a therapeutic target in T-ALL. Doxorubicin is an important chemotherapeutic drug in the treatment of T-ALL and our data suggest that blocking the β1 integrin pathway could enhance its effect and may thus prevent relapse. Deeper knowledge on β1 integrin signaling in T-ALL cells will likely lead to new insights in cell adhesion-mediated drug resistance and to the identification of additional therapeutic targets.

## Materials and methods

### Reagents and antibodies

Growth Factor Reduced Matrigel matrix was purchased from Corning (New York, NY). Doxorubicin and vincristine were from Millipore-Sigma (St-Louis, MO). The PYK2 inhibitor, VS-6063, was purchased from Cayman Chemicals (Ann Arbor, MI). ABCC1 inhibitors, MK571 and reversan, were from Millipore-Sigma (St-Louis, MO) and Tocris-Biotech Canada (Oakville, ON) respectively. The human β1 integrin-specific blocking mAb AIIB2 (rat IgG1), which specifically binds to the β1 integrin extracellular domain^45,46^, was prepared from a hybridoma cell line (Developmental Studies Hybridoma Bank of University of Iowa, Iowa, IA). Its control rat non-specific isotypic antibody was purchased from Santa Cruz Biotechnologies (Santa Cruz, CA). The blocking anti-mouse β1 integrin mAb (Ha2/5) and its related control IgG were purchased from BD Biosciences (San Diego, CA). Anti-phospho PYK2 (Tyr-402) and anti-PYK2 antibodies were purchased from Cell Signaling Technology, Inc (Danvers, MA). Anti-caspase-3 (E-8) recognizing the native and active fragments of caspase-3, and anti-β-actin (C2) antibodies were from Santa Cruz Biotechnology (Santa Cruz, CA). FITC-conjugated anti-human ABCC1 (QCRL-3), PE- and Alexa 647-conjugated anti-human CD5 (UCHT2), the APC-conjugated anti-human β1 integrin (Mar4), control isotypic antibodies and FITC-conjugated Annexin V were purchased from BD Biosciences (San Diego, CA).

### Cell culture and primary blasts

The human T-ALL cell lines Jurkat (E6.1), CCRF-CEM, HSB-2 and Molt-3 were from ATCC (Manassas, VA) and were cultured in RPMI 1640 medium containing 10% of fetal bovine serum, 100 units/ml penicillin and streptomycin, and 2 mmol/l glutamine (complete medium).

T-ALL patients were diagnosed and treated at Hôpital Saint-Louis (Paris, France). The patients or relatives have signed the consent forms according to the Declaration of Helsinki. Hôpital Saint-Louis and Institut Universitaire d’Hématologie Institutional Review Board approved the study. The study was performed with cryopreserved leukemic cells isolated from the blood of three stage IV T-ALL patients at diagnosis.

### Determination of apoptosis and clonogenic survival

T-ALL cells (10^6^/ml) in RPMI 1640 were cultured for 4 h on top of 8 mg/ml of Growth Factor Reduced Matrigel. The medium was then discarded and the cells were treated with doxorubicin in RPMI 1640 complete medium for 24 h. T cell lines and T-ALL primary blasts were treated with 150 and 400 ng/ml of doxorubicin respectively. After drug treatment, apoptosis was analyzed by annexin V staining and flow cytometry using the FACSCalibur cytometer (BD Biosciences).

For clonogenic survival assays, the cells were cultured on plastic or on Matrigel for 4 h and then treated with doxorubicin for 24 h. The cells were recovered, washed and then seeded at 1 × 10^4^ cells/ml in complete medium with 1% methylcellulose (StemCell Technologies, Vancouver, BC). After 21 days, colonies with >50 cells were counted.

### T cell leukemia xenograft and animal protocol

All procedures involving animals were conducted according to the requirements of and with the approval of the Laval University animal protection committee. We used the well-described human T-ALL CEM xenograft model, which involves bone marrow disease and other T-ALL target organs such as the spleen^26,27^. NOD/SCID mice were purchased from Charles River Laboratories (Wilmington, MA) and used at eight weeks old. They were injected i.v. with 10^7^ of CEM cells and five days later, they were randomly divided into four groups and treated for three weeks with control IgG, blocking anti-human β1 integrin mAb AIIB2, IgG + doxorubicin and AIIB2 + doxorubicin. Antibodies (10 mg/kg of body weight) were administered i.p. twice a week, whereas doxorubicin was administered i.p. once a week (1 mg/kg of body weight) and was given one day after the second injection of antibodies. Mice were observed regularly for signs of leukemia and moribund mice were sacrificed. Animal survival was assessed until mice became moribund and was analyzed by generating Kaplan-Meyer curves. Bone marrows were harvested and total cellular suspensions were prepared after red blood lysis. The cellular suspensions were then labeled with anti-human CD5 mAb and analyzed by flow cytometry (FACSCalibur II) for the determination of tumor load.

### Expression of integrins, ABCC1 drug transporter and flow cytometry

The cells were labeled with conjugated control or with specific antibodies against integrins and CD5 for 40 min at 4 °C. For ABCC1 expression, we first permeabilized and fixed the cells with a cytoFix/CytoPerm kit (BD Biosciences) for 30 min at 4 °C, after which, we incubated them with FITC-conjugated isotypic control or anti-ABCC1 antibodies. After antibody staining, the cells were washed and analyzed for integrin, CD5 and ABCC1 expression levels by flow cytometry using BD FACSCalibur II cytometer, and a Canto II flow cytometer (BD Biosciences) for the analysis of β1 integrin on T-ALL blasts.

### Western blot analysis, caspase-3 and PYK2 activation

Activation of caspase-3 and PYK2 was determined by immunoblot analysis as we previously described^47^. After treatment, the cells were harvested, lysed in RIPA buffer and cell lysates subjected to immunoblot analysis using anti-caspase-3 and anti-phospho-PYK2 (Tyr 402) antibodies. The blots were stripped and reprobed with anti-β-actin or anti-PYK2 antibodies for loading control.

### Flow cytometry analysis of intracellular drug content

Jurkat and CEM cells in RPMI 1640 medium were cultured on plastic or Matrigel for 4 h and then treated with doxorubicin in complete RPMI 1640 medium for 2 h at 37 °C. The cells were washed three times with PBS and doxorubicin intracellular content was analyzed by flow cytometry (FACSCalibur; BD Biosciences, San Diego, CA). Doxorubicin fluorescence was detected using the FL-2 channel. The data are presented as the percentage of positive cells times (x) MFI (mean fluorescence intensity) as previously described^48–50^.

### PYK2 siRNA and silencing

Jurkat T cells (5 × 10^6^) were transfected by the Nucleofector method (program C-016; Amaxa Biosystems, Cologne, Germany) with validated siRNA (mix of four sequences) directed against human PYK2 (103485) or with a related non-silencing control siRNA (ThermoFisher Scientific) as we previously described^19^. The cells were then transferred to pre-warmed complete medium and were used 24 h post-transfection in subsequent experiments. The efficiency of PYK2 silencing was assessed by western blot analysis.

### Statistical analysis

Statistical analysis was performed by the Student’s t-test (two-tailed, two samples equal variance). Significance of Kaplan-Meyer survival curves was determined by the log-rank test. *P*-values < 0.05 were considered significant.

## Supplementary information

Supplementary Figure 1.

Supplementary Figure 2.

Supplementary Figure 3.

Supplementary Figure 4.
