# Supplementary figures and images for: Beta1 integrin blockade overcomes doxorubicin resistance in human T-cell acute lymphoblastic leukemia
Source: Cell Death Dis. 2019 May 1;10(5):357. doi: 10.1038/s41419-019-1593-2 (PMC6494825; doi:10.1038/s41419-019-1593-2)

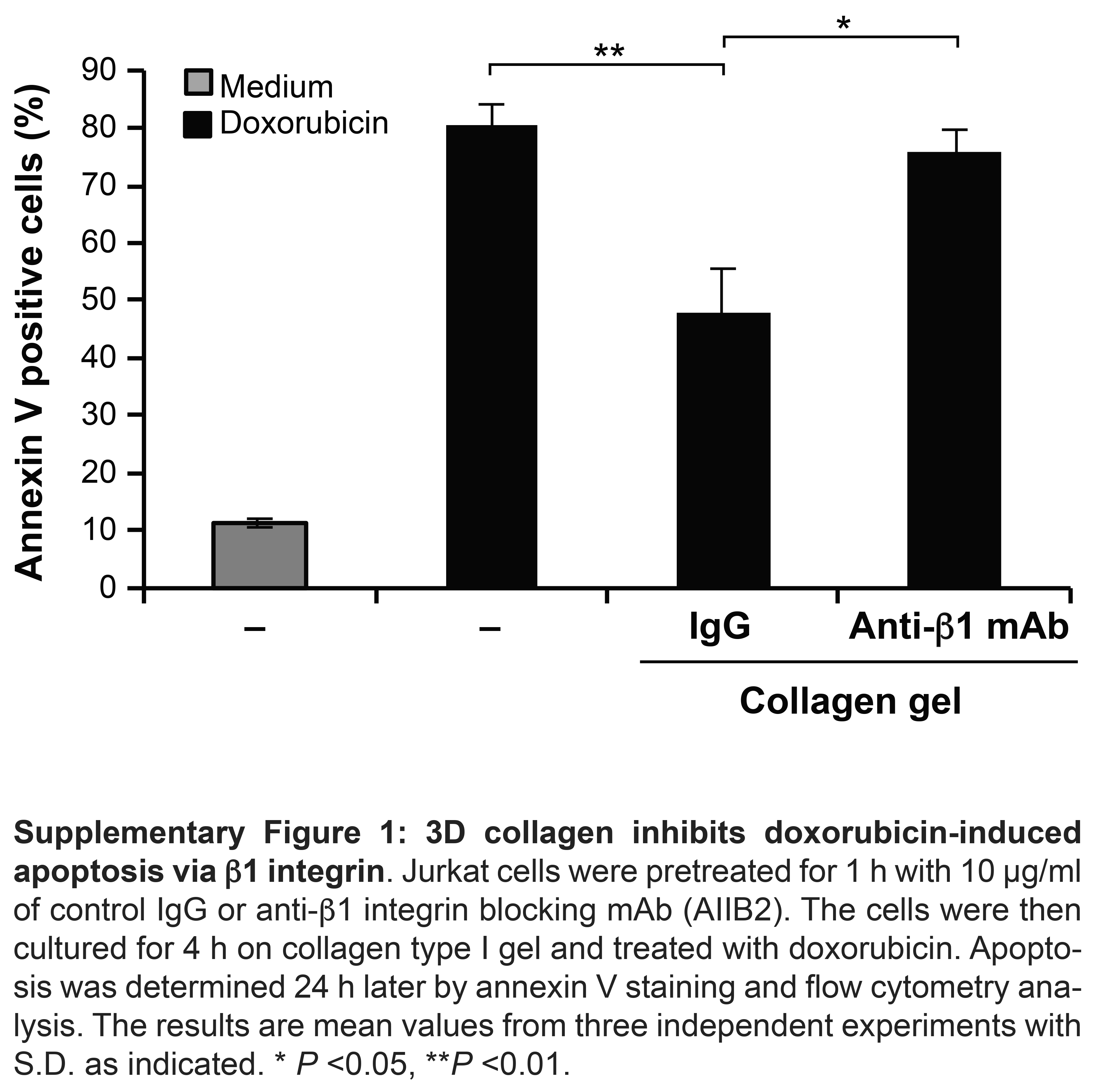

Supplement: Supplementary file 1 — Supplementary Figure 1. [file 41419_2019_1593_MOESM1_ESM.tif]

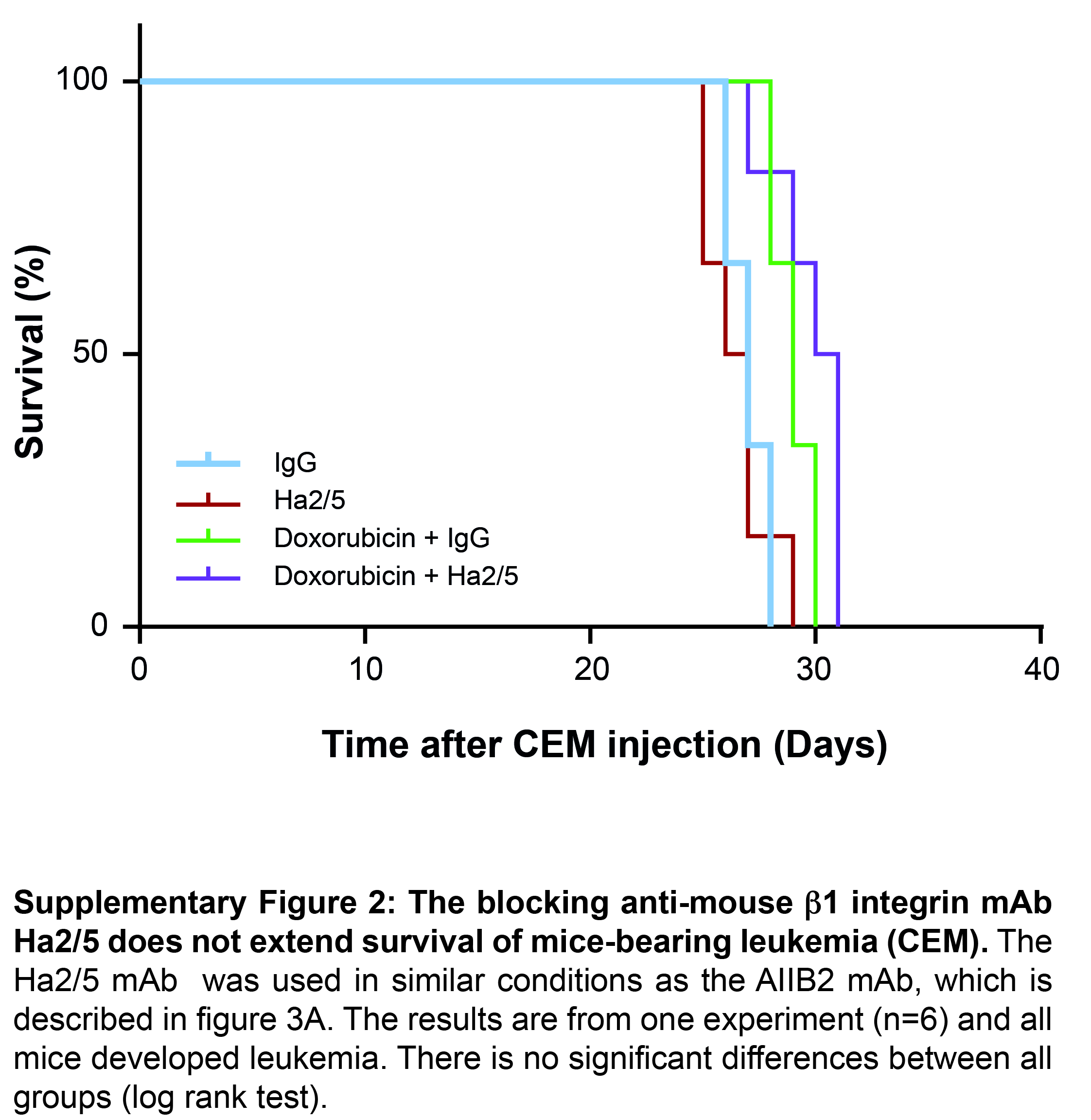

Supplement: Supplementary file 2 — Supplementary Figure 2. [file 41419_2019_1593_MOESM2_ESM.tif]

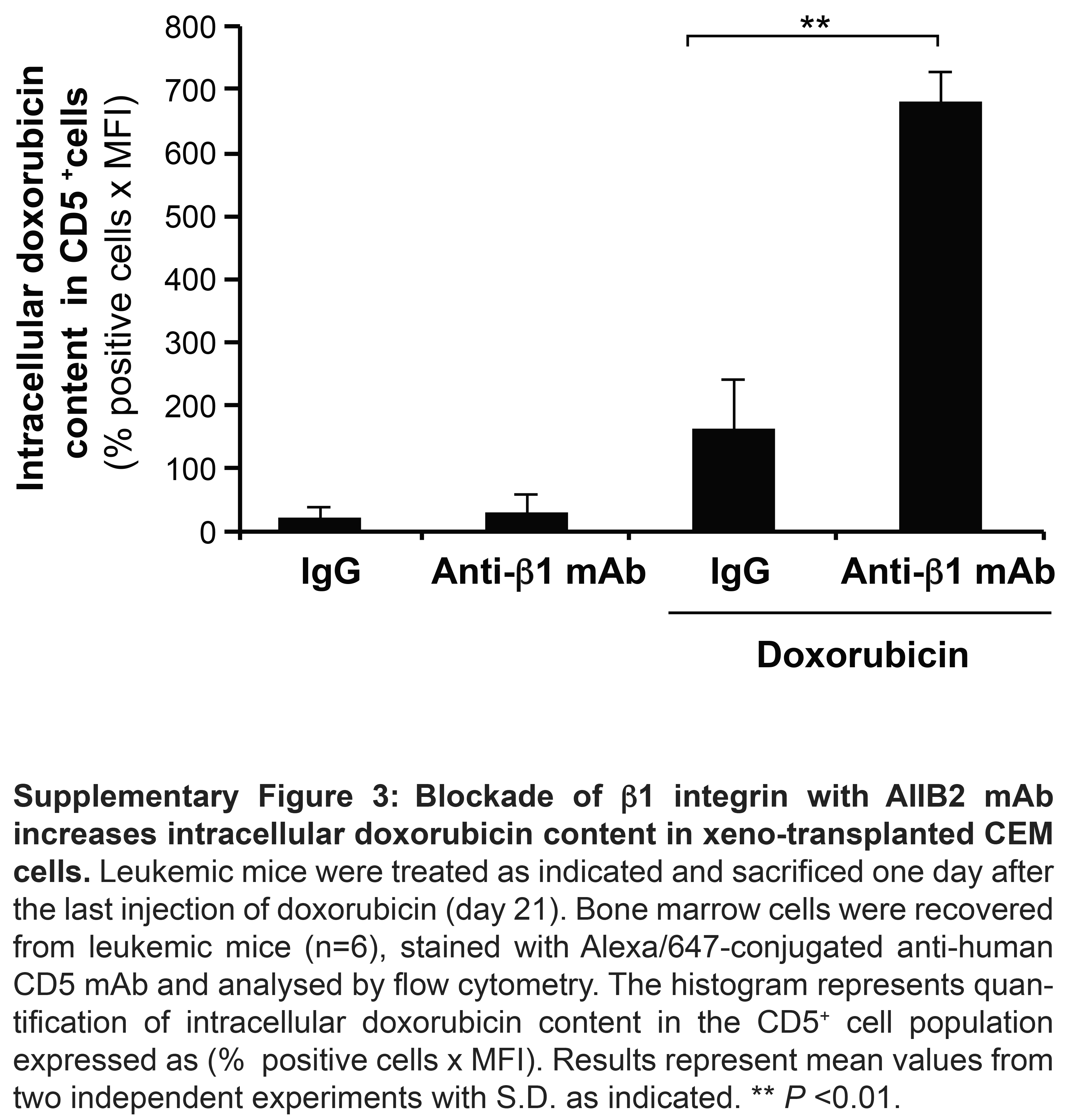

Supplement: Supplementary file 3 — Supplementary Figure 3. [file 41419_2019_1593_MOESM3_ESM.tif]

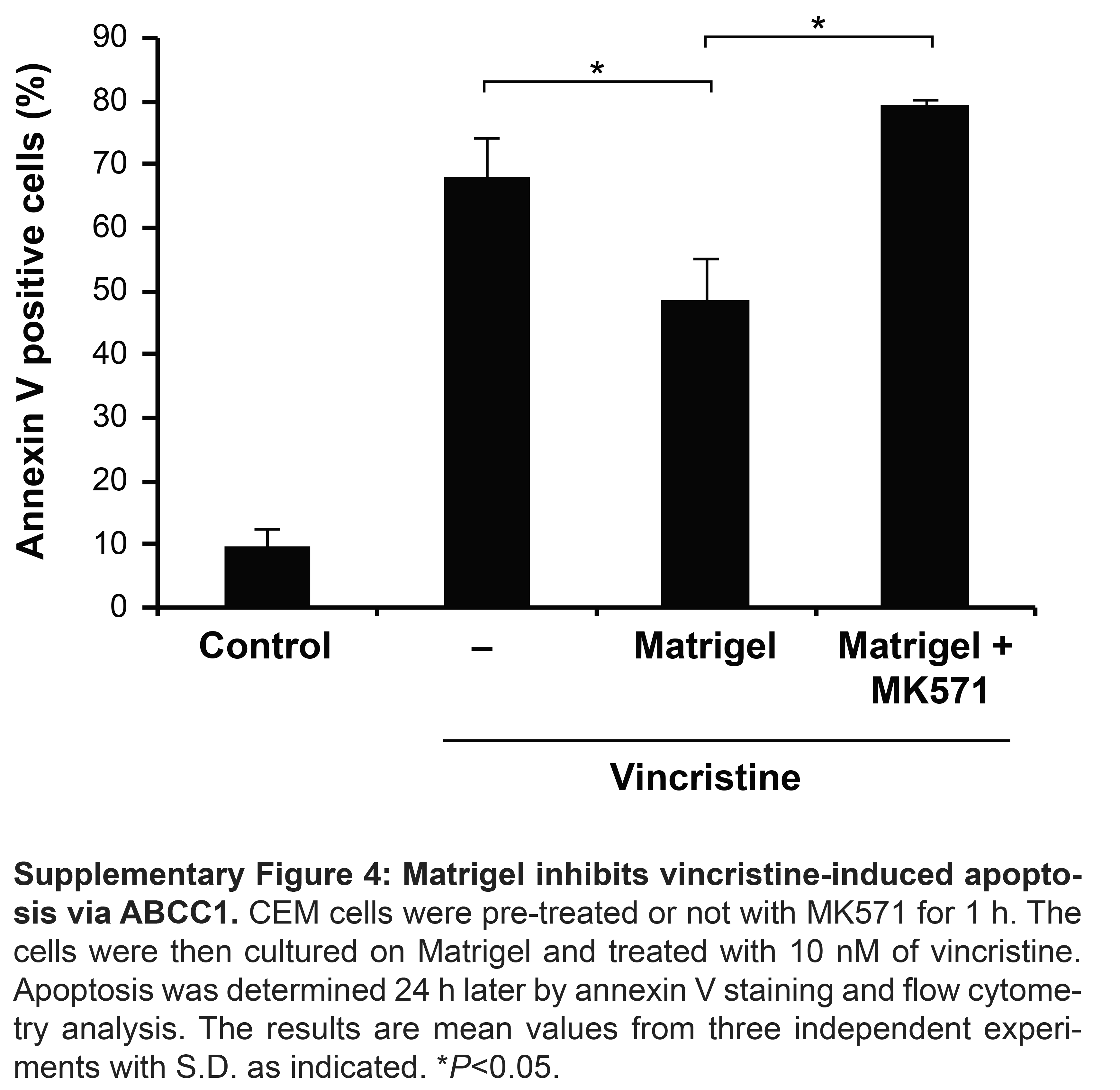

Supplement: Supplementary file 4 — Supplementary Figure 4. [file 41419_2019_1593_MOESM4_ESM.tif]
